# Clinical Evolution of a Cohort of Patients with COVID-19 Treated with Usual Medical Care Plus Polymerized Type I Collagen During the Pandemic Emergency

**DOI:** 10.3390/medsci14010118

**Published:** 2026-03-03

**Authors:** Aurelio Perez-Favila, Idalia Garza-Veloz, Lucia S. Hernandez-Marquez, Maria C. Martinez-Vazquez, Edgar F. Gutierrez-Vela, Ivan Delgado-Enciso, Alfredo Salazar de Santiago, Francisco Luna-Pacheco, Celia E. Luna-Pacheco, Ana G. Castañeda-Miranda, Margarita L. Martinez-Fierro

**Affiliations:** 1 Molecular Medicine Laboratory, Unidad Académica de Medicina Humana y Ciencias de la Salud, Universidad Autónoma de Zacatecas, Zacatecas 98160, Mexico; aurelio.perezf@uaz.edu.mx (A.P.-F.); lucia.hdz@uaz.edu.mx (L.S.H.-M.); calibgst@uaz.edu.mx (M.C.M.-V.); fer_drgtz@yahoo.com.mx (E.F.G.-V.); 2 State Cancerology Institute of Colima, Health Services of the Mexican Social Security Institute for Welfare (IMSS-BIENESTAR), Colima 28085, Mexico; ivan_delgado_enciso@ucol.mx; 3 Unidad Académica de Odontología, Universidad Autónoma de Zacatecas, Zacatecas 98160, Mexico; asalazar@uaz.edu.mx (A.S.d.S.); pacolunap@uaz.edu.mx (F.L.-P.); celialuna@uaz.edu.mx (C.E.L.-P.); 4 Laboratorio de Magnetismo Ambiental, Posgrado en Ingeniería para la Innovación Tecnológica, Unidad Académica de Ingeniería Eléctrica, Universidad Autónoma de Zacatecas, Zacatecas 98000, Mexico; agmiranda@uaz.edu.mx

**Keywords:** COVID-19, polymerized type I collagen, PTIC, SARS-CoV-2 variants

## Abstract

Introduction: The COVID-19 pandemic created an urgent need for safe and accessible outpatient treatments. Polymerized type I collagen (PTIC) has demonstrated potential immunomodulatory effects, but its clinical utility in patients with COVID-19 remains underexplored. This study aimed to evaluate the clinical evolution and health outcomes of a cohort of patients with COVID-19 treated with standard medical care plus PTIC during the pandemic emergency, in order to explore its potential role as an adjuvant therapy. Methods: A retrospective cohort study was conducted in 46 outpatients with confirmed COVID-19 treated with PTIC (Fibroquel^®^) plus standard care, and 15 controls with standard care alone. Clinical and laboratory data were collected on Days 1 and 10. Patients were stratified by COVID-19 severity and SARS-CoV-2 variant. Analyses included odds ratios and Kaplan–Meier survival curves. Results: Oxygen saturation levels increased significantly from 88.5 ± 5.22 to 95.1 ± 2.07 after PTIC treatment. Supplemental oxygen was required for 26.1% of patients receiving treatment, compared to 60% of those in the untreated group (*p* < 0.05). Complete recovery was observed in all patients treated with PTIC, compared with 80% recovery in the standard care group. There were no deaths from COVID-19 in the PTIC group. In contrast, 20% of the participants in the untreated group died due to complications from the disease (*p* = 0.013). PTIC improved the survival rate of patients with the disease. Conclusions: PTIC significantly improved clinical parameters in patients with COVID-19. It improves oxygen saturation levels, decreases the need for supplemental oxygen, and improves survival compared to patients who are not treated with PTIC. Additional studies are needed to validate the use of PTIC as an adjunctive therapy for patients with COVID-19.

## 1. Introduction

The COVID-19 pandemic reshaped healthcare systems and brought to light the wide-ranging effects of the SARS-CoV-2 virus on human health. SARS-CoV-2, the virus responsible for COVID-19, primarily spreads through close contact and airborne droplets, with a secondary risk of transmission via contaminated surfaces [1]. Clinical manifestations vary widely, ranging from mild symptoms such as fever, cough, muscle aches, confusion, and headaches to severe respiratory complications, including acute respiratory distress syndrome (ARDS) [2]. Since emerging in late 2019, the global impact of the pandemic caused by the SARS-CoV-2 has been alarming, with 7,108,060 deaths and 779,059,482 cases worldwide as of December 2025. In Mexico, the pandemic has produced 335,090 deaths and 7,629,738 cases, highlighting its profound local impact [3].

The respiratory system is especially vulnerable to SARS-CoV-2, with many patients developing ARDS and restrictive lung function, sometimes progressing to pulmonary fibrosis [4,5]. Early treatment strategies have included antiviral medications and immunomodulators, though efficacy has varied. While drugs like hydroxychloroquine and chloroquine showed limited success, newer treatments such as Remdesivir and Baricitinib have demonstrated more promising outcomes in severe cases [6,7,8,9,10].

In January 2021, the European Society of Clinical Microbiology and Infectious Diseases (ESCMID) Executive Committee launched a new initiative to develop guidelines on several topics related to the treatment of patients with SARS-CoV-2 infection [11]. The committee recommended the use of monoclonal antibodies such as Bamlanivimab and Etesevimab, and antivirals such as Remdesivir for high-risk outpatients with mild to moderate symptoms. They also found no evidence supporting the continued use of Favipiravir or antifungal prophylaxis and recommended against the routine prescription of antibiotics to patients with suspected or confirmed SARS-CoV-2 infection unless a bacterial coinfection or secondary infection is suspected or confirmed. The use of Tocilizumab and corticosteroids was also suggested for treating severe cases of the disease, but not for mild outpatient cases [12]. Patients with mild COVID-19 (SpO_2_ greater than 93%) do not require hospitalization, either temporary or permanent; however, they should be monitored. High-risk individuals should measure their pulse oximetry at home, and those not at high risk should only receive conservative treatment [13].

Because SARS-CoV-2 is highly infectious, finding effective treatment options became an urgent global priority. In response, numerous therapeutic approaches were investigated during the public health emergency, not only to control the viral infection but also to offer patients alternative or adjunct strategies for symptom management and disease progression [14]. One such molecule is collagen-polyvinylpyrrolidone or polymerized collagen type I (PTIC), a γ-irradiated mixture of pepsinized porcine collagen type I and polyvinylpyrrolidone (PVP), which has been investigated for its potential in managing hyperinflammation, especially in hospitalized patients with COVID-19 [15,16]. Type I collagen has been reported to modulate the leukocyte-associated immunoglobulin-like receptor 1 (LAIR-1 or CD305) and downregulate signal transducer and activator of transcription 1 (STAT-1) phosphorylation, mechanisms of interest in the context of severe inflammatory conditions such as COVID-19 [17]. In line with these efforts to broaden therapeutic options and contribute further scientific evidence, the aim of this study was to evaluate the clinical evolution and health outcomes of a cohort of patients with COVID-19 treated with standard medical care plus PTIC as an adjuvant to standard medical care in a cohort of patients with confirmed COVID-19 who were treated on an outpatient basis during the public health emergency.

## 2. Materials and Methods

### 2.1. Study Design and Setting

A retrospective observational cohort study was conducted to examine the clinical evolution of adult patients with confirmed SARS-CoV-2 infection who received PTIC in addition to standard medical care during the COVID-19 pandemic emergency in Mexico. This study emerged from a clinical follow-up initiative prompted by reports of PTIC use in ambulatory COVID-19 cases. All participants were managed exclusively in outpatient settings, as they declined hospital admission despite physician recommendation. Follow-up data were collected between January 2020 and December 2021 via direct communication with treating physicians in the Zacatecas state. The timeline of recruitment coincided with successive waves of SARS-CoV-2 variants, including Pre-Delta, Delta, and Omicron periods.

### 2.2. Participant Selection

Eligible participants were adults (≥18 years) with a confirmed diagnosis of COVID-19 by RT-PCR, treated either with PTIC (Fibroquel^®^, AspidPharma, Mexico City, Mexico) plus usual medical care or with usual care alone. “Usual medical care” refers to conventional treatment, which was primarily focused on providing symptomatic care and support: corticosteroids and anticoagulation therapy being standard for critically ill patients, in addition to supplemental oxygen as required. For outpatients, treatment was typically limited to paracetamol, monitoring, and rest. Inclusion required complete clinical data at baseline and at day 10 post-treatment initiation. Patients who had received at least one dose of PTIC and had documentation of disease progression were included in the exposed group, while those treated conventionally were included as controls. A total of 61 patients were enrolled in the study: 46 patients received PTIC and constituted the treated cohort and 15 patients who met the same clinical criteria but received only standard care formed the comparison group. Participants with allergies to PTIC, pre-existing lung conditions such as chronic obstructive pulmonary disease (COPD), smokers (cigarettes or vaping), pregnant women, patients with cancer, heart disease, autoimmune disorders, or concurrent infectious diseases other than COVID-19, such as tuberculosis, were not included in the study. Participants who did not adhere to the treatment were excluded. The administration of PTIC was not randomized by the study authors; rather, it was prescribed at the discretion of the treating physicians as adjuvant therapy during the pandemic emergency. The decision to administer PTIC in the exposed group was influenced by individual clinical assessment, drug availability, and patient consent for off-label use.

During the study period, the records of 61 outpatients who met the eligibility criteria were reviewed. All 61 patients were included in the final analysis (100% participation rate) as clinical documentation was available for both baseline (Day 1) and follow-up (Day 10) visits. Consequently, no patients (0%) were lost to follow-up during the study period.

### 2.3. Treatment Protocol and Outcomes

PTIC is a gamma-irradiated mixture of pepsinized porcine collagen type I and a chemical group, polyvinylpyrrolidone (PVP), in a citrate buffer solution. Participants received PTIC (8.33 mg/mL) via intramuscular injection at doses tailored to their COVID-19 severity: 1.5 mL, 2 mL, and 4 mL as a single dose, or in combinations administered every 48 h for up to seven days. Treatment regimens were prescribed at the discretion of each treating physician and adhered to local therapeutic protocols. All patients remained under outpatient surveillance throughout the study period. Data were collected on Day 1 (prior to or at initiation of treatment) and on Day 10. Demographic and clinical background variables, including age, sex, comorbidities, and baseline symptoms, were obtained via physician interviews and verified against medical records. Laboratory tests were ordered as part of routine outpatient management. All data were entered into a standardized electronic form and independently reviewed for consistency and accuracy.

The primary outcome was the clinical evolution of patients during the first 10 days of follow-up, including improvement or worsening of symptoms, oxygen saturation trends, need for supplemental oxygen, and clinical recovery. Secondary outcomes included a panel of laboratory and physiological parameters: hematic biometry (BH), blood chemistry tests, D-dimer, C-reactive protein (CRP), D-hydroxy lactate (DHL), alkaline phosphatase, and ferritin levels. Oxygen saturation and heart rate were also measured. Clinical data were also collected on day 1 and day 10 post-treatment initiation.

### 2.4. COVID-19 Diagnosis and Severity Classification

Diagnosis of COVID-19 relied on common symptoms such as fever, cough, dyspnea, headache, malaise, myalgia, and sore throat, alongside a positive RT-PCR test. Naso/oropharyngeal swab samples were collected and processed using the QIAamp Viral RNA Mini Kit (Qiagen, Hilden, Germany) for RNA extraction. RT-PCR was performed using the TaqMan 2019nCoV assay (Thermo Fisher Scientific, Waltham, MA, USA) according to the manufacturer’s instructions, on the Applied Biosystems StepOne plus thermal cycler (Applied Biosystems, Foster City, CA, USA). Patients were classified based on disease severity according to WHO criteria (Table S1) [18].

### 2.5. Classification of Pre-Delta, Delta and Omicron Variants of SARS-CoV-2

Temporal classification of SARS-CoV-2 variants (Pre-Delta, Delta and Omicron) was done based on the period when each variant accounted for >50% of infections in Mexico, verified via the GISAID platform https://gisaid.org/ (accessed on 26 August 2023). In this sense, the Pre-Delta variant (19 February 2020–7 March 2021), the Delta variant (8 March 2021–25 October 2021) and finally the Omicron variant (26 October 2021–31 December 2021) were identified. Random samples were taken to validate the temporal classification through sequencing using the BigDye Terminator v3.1 Cycle Sequencing commercial kit (Applied Biosystem, Foster, CA, USA).

### 2.6. Bias and Study Size

To mitigate selection bias, all consecutively treated patients meeting the inclusion criteria with available follow-up data were included. Information bias was minimized through direct physician validation and standardized data abstraction protocols. Confounding was partially addressed by stratifying analyses by disease severity and SARS-CoV-2 variant exposure. The sample size was defined pragmatically, based on the number of cases treated with PTIC during the study period for whom complete follow-up data were available. As such, no formal a priori sample size calculation was conducted.

### 2.7. Statistical Analysis

Continuous variables were expressed as mean ± standard deviation and analyzed using the *t*-test or Mann–Whitney U test for significance. Categorical variables were expressed as frequencies and percentages, analyzed using the chi-square test. To assess the association between PTIC treatment and clinical outcomes, Odds Ratios (OR) with 95% confidence intervals (CI) were calculated. Survival analysis was performed using Kaplan–Meier survival curves, and differences between groups were evaluated using the log-rank test. Analyses were stratified by variant period and adjusted for baseline disease severity. All statistical tests were two-sided, and a *p*-value < 0.05 was considered statistically significant. Analyses were performed using Sigma Plot (v14.5) software.

### 2.8. Ethical Considerations

The study was conducted in accordance with the ethical principles established in the Declaration of Helsinki and with applicable national regulations for research involving human subjects. All data were anonymized prior to analysis to ensure confidentiality. As this was a retrospective observational study based on previously collected clinical data and physician follow-up, it did not require direct patient intervention or modification of standard care. Ethical oversight was provided by the Research Ethics Committee of the State Institute of Cancerology (Protocol ID: CEICANCL11122020-COHORTE-12; approval date: 11 December 2020), and informed consent was obtained by treating physicians at the time of PTIC administration, as required for off-label treatments during the pandemic. For research purposes, this retrospective study involved only the analysis of anonymized clinical records and did not include any direct patient intervention. Accordingly, the Ethics Committee granted a waiver of informed consent for participation in the study and publication of its findings. Data use agreements were established to ensure the secure transfer and handling of patient information in compliance with applicable data protection regulations.

## 3. Results

### 3.1. General Findings and Baseline Characteristics

With respect to the integrity of the study, it is noteworthy that all the patients included in the study (*n* = 61) completed the 10-day follow-up. There were no losses to follow-up or dropouts in either the PTIC-treated or conventional therapy group, ensuring the reliability of the comparative data. A total of 61 Mexican patients diagnosed with SARS-CoV-2/COVID-19 were included in the study. 46 participants were included in the PTIC group (patients who received the PTIC drug) and 15 participants in the comparison group (patients with COVID-19 treated with standard therapy). To ensure the groups were comparable, we first analyzed their demographic and clinical characteristics at the time of diagnosis. We found that the groups were well balanced in terms of age and sex distribution. In the PTIC group, 54.3% were women and 45.7% were men, with a mean age of 48.5 ± 12.6 years. In the comparison group, 53.3% were women and 46.7% were men, with an average age of 53.8 ± 13.1 years (*p* > 0.05). Regarding the patients’ clinical presentation, we compared the frequency of symptoms at the time of diagnosis. For this comparison, the chi-square test was applied. The patients in the PTIC group exhibited significantly higher frequency of the following symptoms: cough (86.9% vs. 46.7%), tachycardia (50.0% vs. 13.3%), chest pain (60.9% vs. 6.6%), asthenia (71.7% vs. 20%), adynamia (76.1% vs. 26.6%), myalgia (78.3% vs. 26.6%) and arthralgia (78.3% vs. 26.6%), anosmia (36.9% vs. 6.6%), dysgeusia (36.9% vs. 6.6%), rhinorrhea (63.0 vs. 13.3%), odynophagia (60.9% vs. 6.6%), and general discomfort (76.1% vs. 0%), (*p* < 0.05; Table S2).

The frequency of comorbidities in the study population (Table 1) was as follows: obesity (40.5%), hypertension (23.8%), and type 2 diabetes mellitus (19.0%) in the PTIC group; and obesity (20%), hypertension (46.7%), and type 2 diabetes mellitus (33.3%), in the comparison group. No differences were observed in the frequencies of comorbidities between the study groups (*p* > 0.05).

Laboratory tests showed elevated levels of seven biochemical parameters in the comparison group compared to those observed in the PTIC group. Those parameters were: glucose at diagnosis (160.7 ± 74.5 vs. 106.4 ± 27.2 mg/dL); serum creatinine (1.6 ± 3.7 vs. 0.94 ± 0.27 mg/dL); D-dimer (2426.5 ± 1819.6 vs. 553.3 ± 890.3 ng/mL); and lactate dehydrogenase (900.6 ± 614.26 vs. 327.1 ± 93.7 U/L), hematocrit (59.2% ± 66.0% vs. 46.2% ± 5.6%), leukocytes (9.6 ± 3.9 vs. 6.7 ± 1.5 × 10^3^/μL) and neutrophils (79.9% ± 22.6% vs. 50.1% ± 24.7%), *p* < 0.05 (Table 1). The distribution of disease severity was similar between groups. In the group treated with PTIC, mild, moderate, and severe COVID-19 were observed in 40%, 15%, and 45% of patients, respectively, whereas in the comparison group, these proportions were 40%, 26.7%, and 33.3%, respectively (*p* = 0.538).

### 3.2. Effect of Polymerized Collagen Type I on Clinical Characteristics

**Figure 1:**
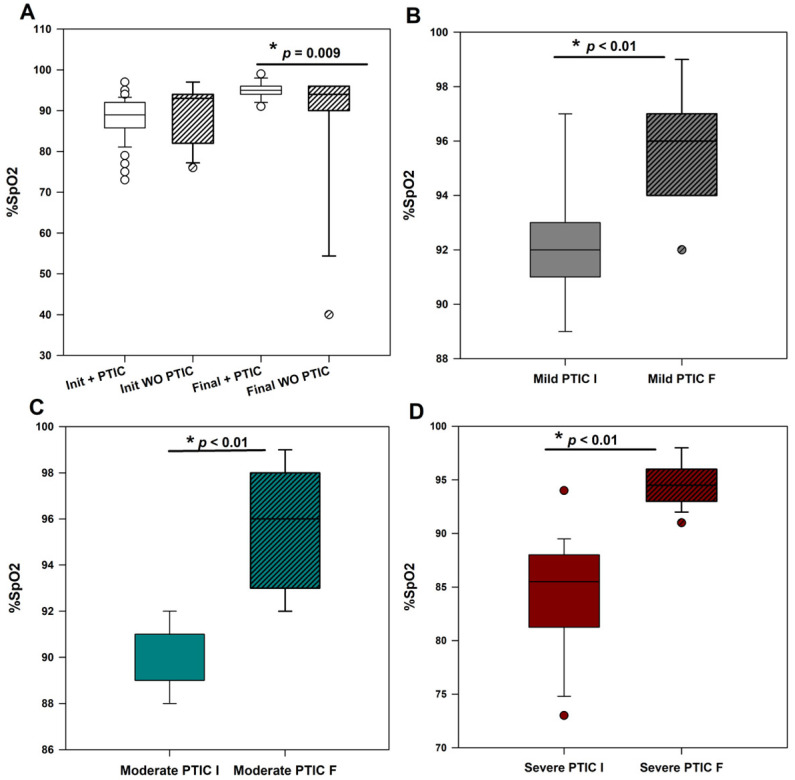
Comparison of the percentage of oxygen saturation (%SpO_2_) between patients receiving PTIC treatment and conventional therapy. (**A**). The initial and final oxygen saturation levels are shown for patients treated with polymerized collagen type 1 (PTIC) and patients who were not treated with the drug (WO = without PTIC). There was a significant improvement in final saturation levels between the two groups, * *p* < 0.05. (**B**–**D**). A subsequent analysis of oxygen saturation was conducted between the treated and untreated groups, with the groups separated according to severity (mild, moderate, and severe). Init = baseline (day 0); Final = day of clinical discharge; PTIC = Polymerized type I collagen group; WO = Without PTIC (control).

In order to ascertain the therapeutic impact of PTIC as an adjunctive therapy, changes in oxygen saturation (%SpO_2_) and the necessity for supplemental oxygen were monitored. A *t*-test was employed to compare oxygen saturation levels before and after the 10-day period. The effect of PTIC on clinical parameters of the participants, particularly oxygen saturation, was favorable (Figure 1). At baseline, the mean oxygen saturation was 88.5% ± 5.22% in the PTIC group versus 89.6% ± 6.9% in the comparison group (*p* > 0.05), improving significantly following treatment to 95.1% ± 2.07% versus 87.9% ± 15.4%, respectively (*p* = 0.009) (Figure 1A).

To determine the consistency of the effect across all clinical stages, oxygen saturation was analyzed separately within the PTIC treatment group at baseline and at the conclusion of treatment. The participants were classified according to their COVID-19 severity (mild, moderate, and severe). An increase in the percentage of SpO_2_ was observed at the conclusion of the treatment in the three comparisons: mild (92.5 ± 2.1 initial vs. 95.6 ± 2.0 final, *p* < 0.001); moderate (89.5 ± 1.3 initial vs. 95.6 ± 2.7 final, *p* < 0.001); and severe (84.3 ± 5.3 initial vs. 94.6 ± 2.1 final, *p* <0.001) (Figure 1B–D) and Table 2.

In a statistical approximation, the participants were classified according to COVID-19 severity (mild, moderate, and severe), and their SpO_2_ levels were compared between study groups (according to their severity), both at the diagnosis and at the end of treatment (Supplementary Figure S1). At the baseline stage, a significant difference was observed between the mild group treated with PTIC and the untreated mild group. The untreated mild group exhibited higher baseline oxygen saturation levels (94.8% ± 1.8 vs. 92.5% ± 2.1; *p* = 0.025). No statistically significant differences were identified in the remaining baseline comparisons (*p* > 0.05). However, upon the conclusion of the PTIC treatment, a discrepancy in oxygen saturation was observed between the two groups: the mild group that received PTIC treatment and the mild group that did not receive any treatment. SpO_2_ levels exhibited an increase in the treated group, with a mean of 95.6% ± 2.0 compared to 93.8% ± 1.3 in the control group (*p* = 0.046). In the other groups of severity, there were no significant differences (*p* > 0.05). A statistical trend toward increased SpO_2_ was observed in the PTIC group after treatment ended (Figure S1).

We compared the proportion of patients requiring oxygen support using a chi-square test. According to the patients’ requirements and their follow-up, during the study, from the PTIC group, 12 of them (26.1%) required supplemental oxygen, compared to 9 patients (60%) in the comparison group (*p* = 0.016; Table 1).

### 3.3. Survival and Clinical Recovery Outcomes

**Figure 2:**
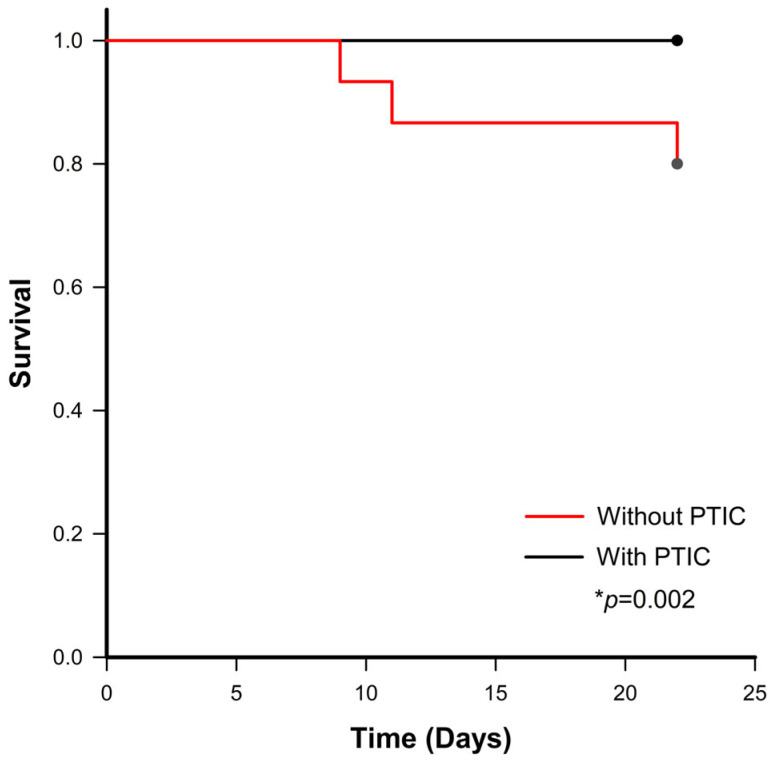
Kaplan–Meier survival curve in patients with and without PTIC treatment. Patients receiving PTIC treatment demonstrated a higher survival rate in comparison to the untreated group, exhibiting a statistically significant difference. PTIC (polymerized collagen type 1). * *p* < 0.05.

To assess the impact of PTIC on patient survival, Kaplan–Meier survival analysis was conducted, and differences between groups were evaluated using the log-rank test. The evaluation of the patient outcome showed the following results: 46 (100%) patients treated with PTIC showed improvement compared to 12 (80%) of those not treated with PTIC. In the same sense, 3 (20%) of the patients not treated with PTIC died due to complications related to the disease (*p* = 0.013). Consequently, treatment with PTIC enhanced survival in comparison to the untreated group (*p* = 0.002; Figure 2).

### 3.4. Prevalence and Risk of Developing Symptoms for Pre-Delta, Delta, and Omicron Variants

In light of the evolving nature of the pandemic, we conducted a thorough analysis to determine whether the infecting variant (Pre-Delta, Delta, or Omicron) influenced the clinical presentation. Among the 61 patients, 20 (32.8%) had the Pre-Delta variant, 21 (34.4%) had the Delta variant, and 20 (32.8%) had the Omicron variant. Patients in the PTIC group were distributed across the Pre-Delta (40%), Delta (85.7%), and Omicron (100%) periods, while those in the control group were concentrated mainly in the Pre-Delta (60%), Delta (14.3%), and Omicron (0%) periods. To assess the prevalence of symptoms, a chi-square test was applied to compare the three groups of variants. Patients with the Omicron variant experienced a higher frequency of several symptoms compared to those with the Delta and Pre-Delta variants. These symptoms included cough (90% vs. 81% and 55%, respectively), asthenia (90% vs. 52.4% and 40%, respectively), adynamia (90% vs. 52.4% and 45%, respectively), myalgia (90% vs. 57.1% and 40%, respectively), arthralgia (90% vs. 57.1% and 40%, respectively), and general discomfort (80% vs. 57.1% and 30%, respectively), *p* < 0.05 (Table 3).

**Figure 3:**
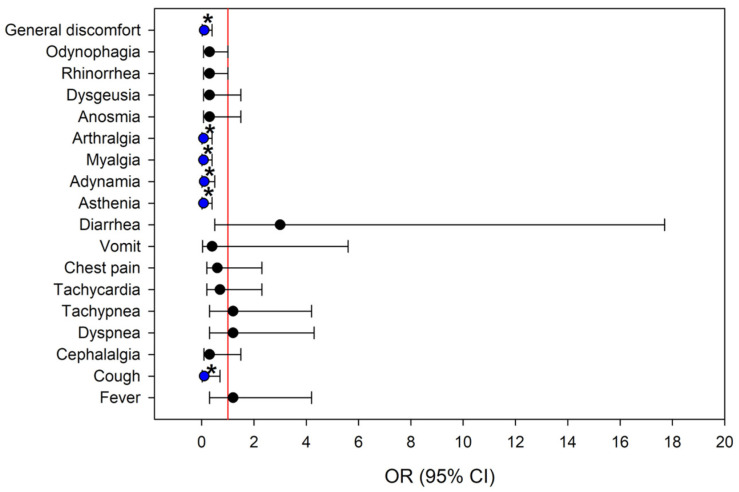
Risk of developing symptoms according to SARS-CoV-2 variants independent of treatment. The figure shows a comparison between Pre-Delta and Omicron. OR indicates Odds Ratio, Upper Control Limit (UCL) and the Lower Control Limit (LCL), blue indicates symptoms that were significant in the odds ratio analysis and had protective values, * *p* < 0.05.

**Figure 4:**
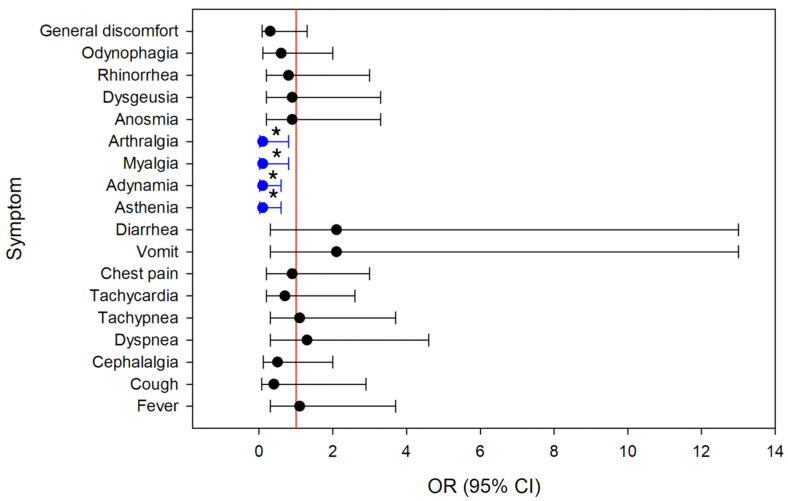
Risk of developing symptoms according to the SARS-CoV-2 variant, independent of treatment. The figure shows the comparison between Delta and Omicron. OR indicates Odds Ratio, Upper Control Limit (UCL) and the Lower Control Limit (LCL), blue indicates symptoms that were significant in the odds ratio analysis and had protective values, * *p* < 0.05.

In order to comprehend the clinical context in which PTIC was administered, an analysis was conducted of the risk of symptom presentation across the different pandemic waves: Pre-Delta, Delta, and Omicron. The ensuing odds ratios (Figure 3 and Figure 4) illustrate the association between the viral variant and the probability of manifesting specific symptoms.

Compared to patients infected with the Omicron variant, those with the Pre-Delta variant exhibited a lower risk of presenting the following symptoms: cough (OR = 0.1; 95% CI: 0.02–0.7; *p* = 0.013), asthenia (OR = 0.07; 95% CI: 0.01–0.4; *p* ≤ 0.001), adynamia (OR = 0.1; 95% CI: 0.01–0.5; *p* = 0.002), myalgia (OR = 0.07; 95% CI: 0.01–0.4; *p* ≤ 0.001), arthralgia (OR = 0.07; 95% CI: 0.01–0.4; *p* ≤ 0.001) and general discomfort (OR = 0.1; 95% CI: 0.02–0.4; *p* = 0.001) (Figure 3).

Compared to the Omicron variant, patients with the Delta variant were found to be at a lower risk of experiencing the following symptoms (Figure 4): asthenia (OR = 0.1; 95% CI: 0.02–0.6; *p* = 0.008), adynamia (OR = 0.1; 95% CI: 0.02–0.6; *p* = 0.008), myalgia (OR = 0.1; 95% CI: 0.02–0.8; *p* = 0.018) and arthralgia (OR = 0.1; 95% CI: 0.02–0.8; *p* = 0.018). The Pre-Delta vs. Delta comparisons did not differ in symptoms (*p* > 0.05). See Table S3.

### 3.5. Adverse Effects

In general, after the administration of the PTIC medication, the adverse effects observed in the patients receiving the treatment were the following: some patients reported experiencing pain after the injection, and others reported a general feeling of freshness in their bodies. Additionally, an increase in blood glucose levels was recorded in the first four days after PTIC application, both in patients with T2DM and in patients without diabetes (no more than 80 mg/dL). Following the four-day period, glycemic control improved to levels even better than those observed before the onset of the respiratory disease.

## 4. Discussion

The emergence of the COVID-19 pandemic emphasized the need for effective treatments, prompting exploration into repurposing existing medications. PTIC is a drug whose initial findings focused on its effect on the regulation of inflammation and clinical improvement in patients with osteoarthritis and arthritis [19,20,21]. A recent report by Mendez-Flores et al. [15] indicated that this drug may offer benefits to patients with COVID-19. These benefits include the modulation of certain markers associated with severe COVID-19 disease, such as IP-10, IL-8, and M-CSF. The aim of this study was to evaluate the clinical evolution, safety profile, and potential therapeutic effect of PTIC administered as an adjunct to standard medical care in a cohort of patients with confirmed COVID-19 who were managed on an outpatient basis during the public health emergency.

One of the principal findings of this study was the effect of PTIC on the clinical signs of oxygen saturation. The SpO_2_ levels in the treated group exhibited a substantial increase, from 88.5% to 95.1%, in contrast to the non-treated group, which demonstrated no clinical improvement in this parameter, but rather a slight decrease from 89.6% to 87.9%. This data is consistent with the findings of Mendez-Flores et al. [15] who observed a similar effect in their study. In their investigation, the value increased from 90% of patients saturating above 92% SpO_2_ to 98% on day eight in the group treated with PTIC. Another research group that evaluated this drug was Carpio et al. [22]. Their findings indicated that PTIC increased %SpO_2_ values, from an initial value of 88% to a value of 94% on day 7 after the commencement of treatment. This suggests that PTIC may be beneficial in clinical improvement, as it increases the values of oxygen saturation.

In this study, the use of PTIC was found to be associated with a reduced need for supplemental oxygen therapy. The findings demonstrated that 12 patients (26.1%) in the PTIC group required supplemental oxygen, while 9 patients (60%) in the untreated group required supplemental oxygen, thereby demonstrating a beneficial effect of this pharmaceutical agent. These data were comparable to those observed by Carpio et al. [22] who observed that at the beginning of the study, before treatment with PTIC, 92% of patients required supplemental oxygen in amounts ranging from 3 to 10 L/min. Seven days after treatment alone, 20% of the patients required supplemental oxygen out of a total of 35 patients of Mexican nationality. This behavior was observed in another study where they evaluated PTIC. In that study, they found that of 87 patients on day 1 after treatment, 6 (7%) needed supplemental oxygen. On day 8 after treatment, 2 of 81 (2.5%) patients required supplemental oxygen. On day 90 after treatment, no patient required supplemental oxygen (0%), although the amounts of supplemental oxygen were not mentioned [15].

**Figure 5:**
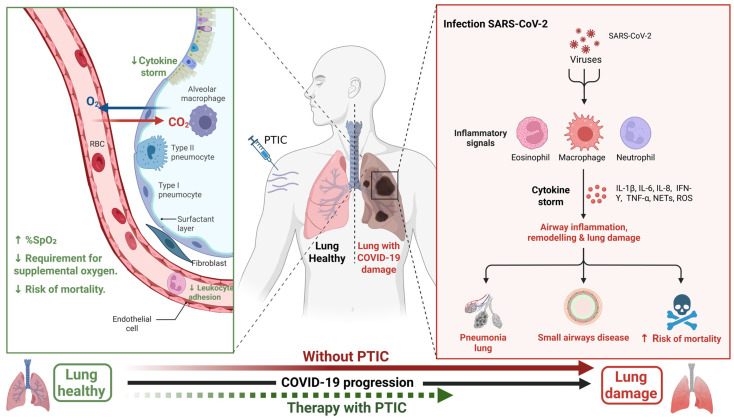
Beneficial effects of PTIC. During the natural progression of SARS-CoV-2 infection, the virus infects lung cells, triggering an immune response involving various cells, including macrophages, neutrophils, and eosinophils. This, in turn, produces the so-called cytokine storm, which can damage lung tissue. PTIC acts by modulating this storm, which is related to inflammation and negative effects on oxygen saturation. Therefore, modulating this phenomenon increases oxygen saturation, decreases the number of patients requiring supplemental oxygen, and decreases the number of patients who die from COVID-19. The black arrow indicates the normal course of the disease without pharmacological intervention. The red arrow indicates the course of the disease observed in our study in patients who did not receive PTIC treatment. It goes from a saline state to lung damage. Finally, the green arrow indicates that the progression to lung damage stops with PTIC use. This helps recover oxygen saturation levels and reduces the risk of death from SARS-CoV-2 infection. The beneficial effects are indicated by the green marks on the left.

A significant positive effect observed in patients treated with PTIC was the reduction in mortality in the group of patients treated with the drug, since no patient in this group died due to complications arising from the disease, while 3 (20%) of the group not treated with this drug died due to complications of the disease. Mortality rates due to disease-related complications vary worldwide, ranging from 0.3% to 5.8% across different countries. In Mexico, mortality has reached 16.2% [23], a figure very close to that observed in the study population (20%). Two meta-analyses have yielded results that are analogous to those obtained in the present study. As reported by Baptista et al. [24] the mortality rate was of 26%. In contrast, Dessie et al. [25] reported a mortality rate of 17.62% in patients who had been hospitalized. It has been demonstrated by several studies that a variety of factors have the potential to elevate the risk of mortality due to COVID-19, including age, sex, and the existence of comorbidities and disease severity [26]. This study did not find significant differences between the PTIC-treated and controls with respect to age, sex, the presence of comorbidities, or disease severity. The enhanced survival rate may be ascribed to the utilization of PTIC. According to the above and accumulated evidence, in Figure 5, we summarize the beneficial effects of PTIC.

The beneficial effects of PTIC could be attributed to the modulation of pro-inflammatory cytokines, as previously reported by Mendez-Flores et al. [15] who found a decrease in IP-10, IL-8 and M-CSF, which are markers of severe disease. Additionally, they observed a significant decrease in hsCRP, D-dimer and LDH, which are also biomarkers related to both activity and severity of COVID-19. The modulation of cytokines was also observed by Furuzawa et al. [17] They reported that on day one after treatment with PTIC, in the female group, there was a decrease in the cytokines IL-1Ra, IL-8, and IP-10, which are associated with inflammation in cases of severe COVID-19. In contrast, in the male group, SCGF-β and IP-10 increased, while IL-1Ra and IL-8 decreased. Another study reported similar data, indicating that PTIC decreased the percentage of macrophages and M1 cytokines (IP-10, MIF, eotaxin, IL-8, IL-1Ra, and M-CSF). This decrease was directly related to an improvement in oxygen saturation, in addition to a reduction in symptoms such as dyspnea, cough, chest pain, and chronic fatigue syndrome [27]. These data provide an explanation for the early recovery observed in patients treated with PTIC in the study group. Finally, in the population under evaluation, an elevation of blood glucose was recorded in the first four days after PTIC application, in patients with T2DM and in patients without diabetes. However, other authors report that no serious adverse effects have been found and that PTIC use is safe and well tolerated by patients, as reported by Mendez-Flores et al. and Olivares-Martínez et al. [15,27]. These authors also mention the safety of the drug, reporting pain at the injection site and no serious adverse effects.

In our study population, the risk of presenting symptoms associated with the variants was as follows: compared to Omicron, the Pre-Delta variant was found to be at a lower risk of experiencing cough, asthenia, adynamia, myalgia, arthralgia, and general discomfort. The Delta variant was found to be at a lower risk of experiencing asthenia, adynamia, myalgia, and arthralgia compared to Omicron. This pattern differed from that reported by other authors. Günther et al. [28] found that patients infected with the Delta variant were more likely to experience fever, myalgia, fatigue, and malaise than those infected with the wild-type/alpha variant. However, they also found that the odds of experiencing myalgia and fatigue were significantly higher in patients infected with the Omicron variant than in those infected with the wild-type/alpha variant. In another study, Sumner et al. [29] reported that both Omicron and Delta infections were associated with fever and cough. Delta infections were associated with upper respiratory tract symptoms, while Omicron infections were associated with lower respiratory tract symptoms and systemic symptoms. Anosmia, ageusia, and rash or oral changes were less strongly associated with the Alpha and Omicron strains than with the original strain. These shifts may reflect the evolving impact of the virus on different organ systems. Newer variants may affect the respiratory tract more prominently, causing upper respiratory symptoms rather than systemic symptoms such as fever or severe dyspnea [30]. Together, these findings highlight the dynamic nature of SARS-CoV-2 symptomatology and show the importance of continuously adapting clinical management to variant-specific presentations.

It is imperative to acknowledge the limitations of this study when interpreting the findings. Firstly, the study design was retrospective and non-randomized, which introduces an inherent risk of selection bias due to the context of a healthcare emergency. The decision to administer PTIC was predicated on clinical criteria and the availability of patients. Secondly, the sample size was not predetermined, and there was a significant numerical imbalance between the treated and control groups. Thirdly, the baseline biochemical markers indicated a heightened inflammatory state in the control group, which may be a contributing factor to the observed disparities in mortality. Despite these limitations, the results provide evidence of the potential benefits of PTIC as an adjuvant therapy in cases of moderate to severe COVID-19. Future research should include prospective, randomized controlled trials with adequately powered sample sizes and balanced severity strata, and multivariate analyses to validate these observations. Additionally, standardized treatment protocols, longer follow-up periods, and stratified analyses by disease severity and viral variant will be essential to confirm efficacy, assess safety, and better define the clinical role of PTIC in COVID-19 and related inflammatory conditions.

## 5. Conclusions

PTIC improved clinical parameters in patients with COVID-19. The first effect observed in the study population was a significant increase in oxygen saturation percentage, rising from 88.5 ± 5.22 to 95.1 ± 2.07 after treatment. Second, the percentage of patients requiring supplemental oxygen decreased, from 60% in the untreated group to 26.1% in the PTIC treatment group. Finally, PTIC affected survival: none of the patients treated with the drug died from COVID-19, while 20% of untreated patients died from it. These results suggest that PTIC could be a beneficial alternative for adjuvant treatment of patients with SARS-CoV-2. Furthermore, data on SARS-CoV-2 variants and their impact on symptom presentation suggest that these variants may influence both the risk and frequency of symptoms in the study population; therefore, the proposal of new therapeutic strategies remains necessary. Additional studies are needed to validate the use of PTIC as an adjunctive therapy for patients with COVID-19.

## Figures and Tables

**Table 1:** Baseline comorbidities and laboratory parameters of the study groups at the time of diagnosis.

Finding	COVID-19 Cases Treated with PTIC (*n* = 42 ^†^)	COVID-19 Cases Treated with Conventional Therapy (*n* = 15)	*p*-Value
Type 2 diabetes mellitus *n* (%)	8 (19.0)	5 (33.3)	0.294
Hypertension *n* (%)	10 (23.8)	7 (46.7)	0.113
Obesity *n* (%)	17 (40.5)	3 (20)	0.154
Other comorbidities *n* (%)	7 (16.7)	0 (0)	-
Requirements of supplemental oxygen, *n* (%)	12 (26.1)	9 (60)	0.016 *
Glucose (mg/dL)	106.4 ± 27.2	160.7 ± 74.5	0.001 *
Total cholesterol (mg/dL)	174.3 ± 34.5	161.2 ± 59.3	0.525
Triglycerides (mg/dL)	140.1 ± 45.2	155.5 ± 22.5	0.442
Uric acid (mg/dL)	4.3 ± 1.1	4.3 ± 1.3	0.988
Serum creatinine (mg/dL)	0.94 ± 0.27	1.6 ± 3.7	0.013 *
Urea (mg/dL)	32.1 ± 11.1	48.4 ± 51.3	0.287
Blood urea nitrogen (mg/dL)	15.6 ± 5.4	14.7 ± 4.1	0.909
Erythrocyte Sedimentation Rate (mm/h)	24 ± 16.1	19.3 ± 8.5	0.699
D-dimer (ng/mL)	553.3 ± 890.3	2426.5 ± 1819.6	0.017 *
Lactate dehydrogenase (U/L)	327.1 ± 93.7	900.6 ± 614.26	0.033 *
C Reactive Protein (mg/L)	89.3 ± 53.7	165 ± 63.4	0.132
Alkaline phosphatase (IU/L)	99.4 ± 22.3	NA	NA
Serum ferritin (ng/mL)	242.3 ± 214.5	NA	NA
Hemoglobin (g/dL)	15.2 ± 2.0	14.2 ± 3.4	0.098
Hematocrit (%)	46.2 ± 5.6	59.2 ± 66.0	0.029 *
Mean corpuscular volume (fL)	88.0 ± 6.4	85.9 ± 7.1	0.467
Mean corpuscular hemoglobin (pg/cell)	28.9 ± 2.6	28.5 ± 3.3	0.78
Mean corpuscular hemoglobin concentration (g/dL)	32.9 ± 2.9	33.2 ± 1.5	0.811
Red Cell Distribution (%)	15.2 ± 7.8	15.4 ± 2.4	0.095
Platelets (10^3^/μL)	251.3 ± 73.0	252.7 ± 122.8	0.97
Leukocytes (10^3^/μL)	6.7 ± 1.5	9.6 ± 3.9	0.009 *
Lymphocytes (%)	23.6 ± 14.0	14.6 ± 19.9	0.108
Neutrophils (%)	50.1 ± 24.7	79.9 ± 22.6	<0.001 *

Laboratory data are represented as mean ± standard deviation. * *p* < 0.05. Data at the time of COVID-19 diagnosis. ^†^ Laboratory data were only obtained from 42 patients in the PTIC group. NA: data not available.

**Table 2:** Oxygen saturation and supplemental oxygen therapy in patients with PTIC and conventional therapy.

Variable	PTIC Group (*n* = 46)	Comparison Group (*n* = 15)	*p*-Value
Initial O_2_ saturation %, Mean ± SD	88.5 ± 5.22	89.6 ± 6.9	0.271
Final O_2_ saturation %, Mean ± SD	95.1 ± 2.07	87.9 ± 15.4	0.009 *
COVID-19 severity	PTIC group (Initial saturation %, mean ± SD)	PTIC group (Final saturation %, Mean ± SD)	*p*-Value
Mild	92.5 ± 2.1	95.6 ± 2.0	<0.001 *
Moderate	89.5 ± 1.3	95.6 ± 2.7	<0.001 *
Severe	84.3 ± 5.3	94.6 ± 2.1	<0.001 *

* Statistical significance indicated at *p* < 0.05.

**Table 3:** Prevalence of symptoms for each SARS-CoV-2 variant.

Symptoms *n* (%)	Pre-Delta (*n* = 20)	Delta (*n* = 21)	Omicron (*n* = 20)	*p*-Value
Fever	11 (55)	11 (52.4)	10 (50)	0.951
Cough	11 (55)	17 (81)	18 (90)	0.028 *
Headache	12 (60)	14 (66.7)	16 (80)	0.38
Dyspnea	12 (60)	13 (61.9)	11 (55)	0.899
Tachypnea	10 (50)	10 (47.6)	9 (45)	0.951
Tachycardia	7 (35)	8 (38.1)	9 (45)	0.803
Chest pain	8 (40)	10 (47.6)	10 (50)	0.802
Vomit	1 (5)	4 (19)	2 (10)	0.358
Diarrhea	5 (25)	4 (19)	2 (10)	0.462
Asthenia	8 (40)	11 (52.4)	18 (90)	0.003 *
Adynamia	9 (45)	11 (52.4)	18 (90)	0.007 *
Myalgia	8 (40)	12 (57.1)	18 (90)	0.004 *
Arthralgia	8 (40)	12 (57.1)	18 (90)	0.004 *
Anosmia	3 (15)	7 (33.3)	7 (35)	0.291
Dysgeusia	3 (15)	7 (33.3)	7 (35)	0.291
Rhinorrhea	6 (30)	12 (57.1)	12 (60)	0.11
Odynophagia	6 (30)	10 (47.6)	12 (60)	0.16
General discomfort	6 (30)	12 (57.1)	16 (80)	0.006 *

* Statistical significance was defined as *p* < 0.05 in the comparison of symptom frequency among SARS-CoV-2 variants using the chi-square test.

## Data Availability

The data presented in this study are available on request from the corresponding author. (The data that support the findings of this study are available from the corresponding authors upon reasonable request and justification, in accordance with institutional and ethical data protection policies).

## Supplementary Materials

The following supporting information can be downloaded at: https://www.mdpi.com/article/10.3390/medsci14010118/s1, Table S1. Gravity of COVID-19 on adults. WHO criteria; Table S2. Comparison of symptoms at diagnosis in COVID-19 patients with and without treatment; Table S3. Comparison between the presence of SARS-CoV-2 variants and the risk of presenting COVID-19 symptoms; Figure S1. Comparisons in oxygen saturation in the PTIC and non-PTIC groups according to COVID-19 severity.

## Abbreviations

The following abbreviations are used in this manuscript:

PTIC	Polymerized type I collagen
SARS-CoV-2	Severe acute respiratory syndrome coronavirus 2
ARDS	Acute respiratory distress syndrome
ESCMID	European Society of Clinical Microbiology and Infectious Diseases
PVP	polyvinylpyrrolidone
SpO_2_	Blood oxygen saturation
BH	Blood hemoglobin
CRP	C-reactive protein
DHL	D-hydroxy lactate
WHO	World Health Organization
OR	Odds Ratio
UCL	Upper Control Limit
LCL	Lower Control Limit
LAIR-1	Leukocyte-associated immunoglobulin-like receptor 1
COPD	Chronic obstructive pulmonary disease
CI	Confidence intervals
RNA	Ribonucleic acid
T2DM	Type 2 Diabetes Mellitus
IP-10	Interferon-gamma-inducible protein 10
IL-8	Interleukin-8
M-CSF	Macrophage Colony-Stimulating Factor
hsCRP	High-sensitivity C-reactive protein
IL-1Ra	Interleukin-1 Receptor Antagonist
SCGF-β	Stem Cell Growth Factor-beta
MIF	Macrophage Migration Inhibitory Factor
