# Supplementary material for: Clinical Evolution of a Cohort of Patients with COVID-19 Treated with Usual Medical Care Plus Polymerized Type I Collagen During the Pandemic Emergency
Source: Med Sci (Basel). 2026 Mar 3;14(1):118. doi: 10.3390/medsci14010118 (PMC13027494; doi:10.3390/medsci14010118)
Supplement: Supplementary file 1 [file medsci-14-00118-s001.zip › Figure Supplementary material vf.pdf]

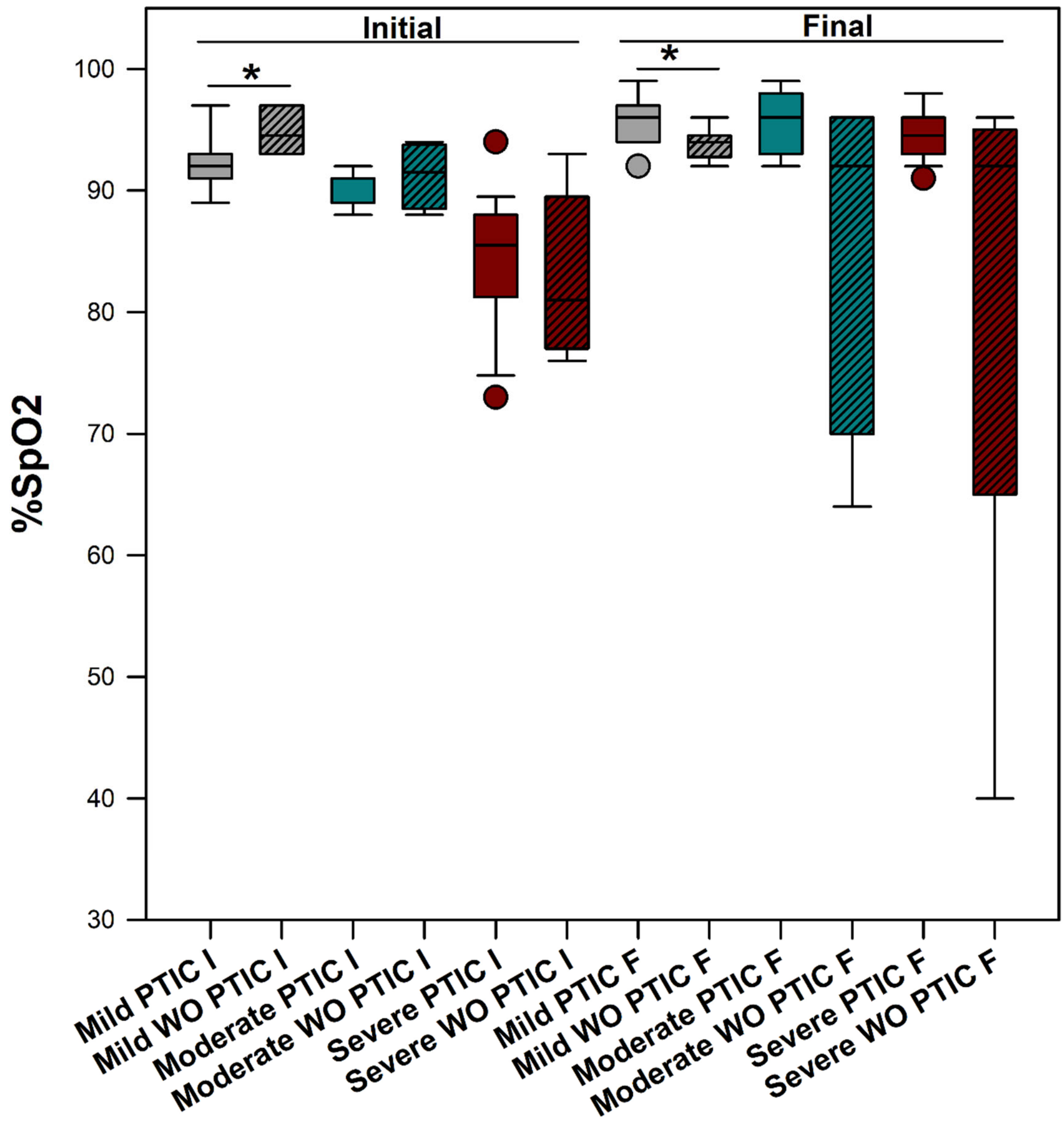

Figure S1. Comparisons in oxygen saturation in the PTIC and non-PTIC groups according COVID-19 severity. There was difference in oxygen saturation between the mild groups with and without PTIC at the initial stage. The group that did not receive treatment exhibited higher levels of oxygen saturation (94.8% ± 1.8 vs. 92.5% ± 2.1, $p = 0.025$). No statistically significant differences were identified in the remaining comparisons at the initial time point ($p > 0.05$). At the conclusion of the treatment period, a difference was observed among the groups previously mentioned: mild cases with PTIC versus mild cases without treatment. However, an enhancement in saturation was observed in the PTIC treatment group, with values of 95.6% ± 2.0 as compared to 93.8% ± 1.3 ($p = 0.046$). In the other groups, no significant differences were observed ($p > 0.05$). Initial = baseline (day 0); Final = day of clinical discharge; PTIC = Polymerized type I collagen group; WO = Without PTIC (control). *$p < 0.05$.
