# Supplementary material for: Clinical Evolution of a Cohort of Patients with COVID-19 Treated with Usual Medical Care Plus Polymerized Type I Collagen During the Pandemic Emergency
Source: Med Sci (Basel). 2026 Mar 3;14(1):118. doi: 10.3390/medsci14010118 (PMC13027494; doi:10.3390/medsci14010118)
Supplement: Supplementary file 1 [file medsci-14-00118-s001.zip › Tables Supplementary material vf.pdf]

Table S1. Gravity of COVID-19 on adults. WHO criteria [18].

Mild Disease		Symptomatic person, without pneumonia and hypoxemia.
Moderate Disease	Pneumonia	Manifestation clinics of pneumonia (fever, cough, dyspnea, tachypnea), $SpO2 \geq 90\%$ on room air without signs of severe pneumonia
Severe Disease	Severe Pneumonia	Manifestation clinics of pneumonia (fever, cough, dyspnea, tachypnea) plus one of the following: respiratory rate > 30 breaths/min, distress respiratory severe $SpO2 < 90\%$ room air
Critical Disease	Syndrome of difficulty respiratory acute (ARDS)	Thoracic radiology: bilateral opacities not fully explained by volume overload, lobar or pulmonary collapse, or nodules<br>Origin of pulmonary infiltrates: respiratory failure not fully explained by heart failure or fluid overload.<br>Poor oxygenation:<br>• Mild ARDS: 200 mm Hg < PaO2/FiO2 to ≤ 300 mm Hg (with PEEP or CPAP ≥ 5 cm H2O).<br>• Moderate ARDS: 100 mm Hg < PaO2/FiO2 ≤ 200 mmHg (with PEEP ≥ 5 cm H2O).<br>• Severe ARDS: PaO2/FiO2 ≤ 100 mm Hg (with PEEP ≥ 5 cm H2O
	Septicemia	Acute and life-threatening organ dysfunction caused by a dysregulation of the host response to suspected or proven infection. Signs of organ dysfunction: altered mental status, dyspnea or tachypnea, low SpO2, oliguria, tachycardia, weak pulse, cold extremities or low blood pressure, mottled skin, evidence of coagulopathy on laboratory tests, thrombocytopenia, acidosis, hyperlactatemia, or hyperbilirubinemia.
	Septic shock	Serum lactate > 2 mmol/l and persistent hypotension which, despite volume replacement, needs vasopressors to maintain a mean BP ≥ 65 mm Hg

Abbreviations: SpO2, oxygen saturation; PEEP, positive end-expiratory pressure; CPAP, continuous positive airway pressure; H2O, water; BP, blood pressure; PaO2/FiO2, is the ratio of arterial oxygen partial pressure (PaO2 in mmHg) to fractional inspired oxygen (FiO2 expressed as a fraction, not a percentage).

Reference: [18]. WHO. Clinical Management of COVID-19: Living Guideline, World Health Organization: Geneva, Switzerland, 2022. Available online: https://apps.who.int/iris/bitstream/handle/10665/362783/WHO-2019-nCoV-Clinical-2022.2-eng.pdf (accessed on 06 June 2025).

Table S2. Comparison of symptoms at diagnosis in COVID-19 patients with and without treatment.

Variable	COVID-19 cases treated with Fibroquel (*n*= 46)	COVID-19 cases treated with conventional therapy (*n*= 15)	*p*-Value
**Sex *n* (%)**			
Male	21 (45.7)	7 (46.7)	0.945
Female	25 (54.3)	8 (53.3)	
**Age (years)**	48.5 ± 12.6	53.8 ± 13.1	0.181
Symptoms	*n* (%)	*n* (%)	
Fever	26 (56.5)	7 (46.7)	0.506
Cough	40 (86.9)	8 (46.7)	0.011*
Cephalalgia	36 (78.2)	9 (46.7)	0.188
Dyspnea	28 (60.8)	9 (60)	0.952
Tachypnea	24 (52.2)	6 (40)	0.330
Tachycardia	23 (50)	2 (13.3)	0.012*
Chest pain	28 (60.9)	1 (6.6)	<0.001*
Vomit	7 (15.2)	1 (6.6)	0.666
Diarrhea	8 (17.4)	4 (26.6)	0.467
Asthenia	33 (71.7)	3 (20)	<0.001*
Adynamia	35 (76.1)	4 (26.6)	<0.001*
Myalgia	36 (78.3)	4 (26.6)	<0.001*
Arthralgia	36 (78.3)	4 (26.6)	<0.001*
Anosmia	17 (36.9)	1 (6.6)	0.047*
Dysgeusia	17 (36.9)	1 (6.6)	0.047*
Rhinorrhea	29 (63)	2 (13.3)	<0.001*
Odynophagia	28 (60.9)	1 (6.6)	<0.001*
General discomfort	35 (76.1)	0 (0)	<0.001*

* Statistical significance indicated at $p < 0.05$.

Table S3. Comparison between the presence of SARS-CoV-2 variants and the risk of presenting COVID-19 symptoms.

Symptoms	Pre-Delta vs Delta		Pre-Delta vs Omicron		Delta vs Omicron	
	OR (95% CI)	p-Value	OR (95% CI)	p-Value	OR (95% CI)	p-Value
Fever	1.1 (0.3-3.7)	0.867	1.2 (0.3-4.2)	0.752	1.1 (0.3-3.7)	0.879
Cough	0.3 (0.07-1.1)	0.074	0.1 (0.02-0.7)	0.013*	0.4 (0.07-2.9)	0.663
Cephalalgia	0.7 (0.2-2.6)	0.658	0.3 (0.09-1.5)	0.168	0.5 (0.12-2.0)	0.335
Dyspnea	0.9 (0.2-3.2)	0.923	1.2 (0.3-4.3)	0.749	1.3 (0.3-4.6)	0.654
Tachypnea	1.1 (0.3-3.7)	0.879	1.2 (0.3-4.2)	0.752	1.1 (0.3-3.7)	0.867
Tachycardia	0.8 (0.2-3.1)	0.837	0.7 (0.2-2.3)	0.519	0.7 (0.2-2.6)	0.654
Chest pain	0.7 (0.2-2.5)	0.623	0.6 (0.2-2.3)	0.525	0.9 (0.2-3.0)	0.879
Vomit	0.2 (0.02-2.2)	0.343	0.4 (0.03-5.6)	1	2.1 (0.3-13.0)	0.663
Diarrhea	1.4 (0.3-6.2)	0.719	3.0 (0.5-17.7)	0.407	2.1 (0.3-13.0)	0.663
Asthenia	0.6 (0.2-2.0)	0.427	0.07 (0.01-0.4)	<0.001*	0.1 (0.02-0.6)	0.008*
Adynamia	0.7 (0.2-2.5)	0.636	0.1 (0.01-0.5)	0.002*	0.1 (0.02-0.6)	0.008*
Myalgia	0.5 (0.1-1.7)	0.272	0.07 (0.01-0.4)	<0.001*	0.1 (0.02-0.8)	0.018*
Arthralgia	0.5 (0.1-1.7)	0.272	0.07 (0.01-0.4)	<0.001*	0.1 (0.02-0.8)	0.018*
Anosmia	0.3 (0.07-1.6)	0.277	0.3 (0.07-1.5)	0.144	0.9 (0.2-3.3)	0.91
Dysgeusia	0.3 (0.07-1.6)	0.277	0.3 (0.07-1.5)	0.144	0.9 (0.2-3.3)	0.91
Rhinorrhea	0.3 (0.08-1.1)	0.08	0.3 (0.07-1.0)	0.057	0.8 (0.2-3)	0.853
Odynophagia	0.4 (0.1-1.7)	0.248	0.3 (0.07-1.0)	0.057	0.6 (0.1-2.0)	0.427
General discomfort	0.3 (0.08-1.1)	0.08	0.1 (0.02-0.4)	0.001*	0.3 (0.08-1.3)	0.116

* Statistical significance indicated at $p < 0.05$.
